# Diversity and Inheritance of Intergenic Spacer Sequences of 45S Ribosomal DNA among Accessions of *Brassica oleracea* L. var. *capitata*

**DOI:** 10.3390/ijms161226125

**Published:** 2015-12-03

**Authors:** Kiwoung Yang, Arif Hasan Khan Robin, Go-Eun Yi, Jonghoon Lee, Mi-Young Chung, Tae-Jin Yang, Ill-Sup Nou

**Affiliations:** 1 Department of Horticulture, Sunchon National University, Suncheon 540-950, Korea; ykw7685@naver.com (K.Y.); gpb21bau@gmail.com (A.H.K.R.); yeege91@hanmail.net (G.-E.Y.); 2 Department of Plant Science, Seoul National University, Seoul 151-742, Korea; jonghoonlee@snu.ac.kr; 3 Department of Agricultural Education, Sunchon National University, Suncheon 540-950, Korea; queen@sunchon.ac.kr

**Keywords:** ribosomal DNA, intergenic spacer, 45S rDNA, *Brassica oleracea* var. *capitata*

## Abstract

Ribosomal DNA (rDNA) of plants is present in high copy number and shows variation between and within species in the length of the intergenic spacer (IGS). The 45S rDNA of flowering plants includes the 5.8S, 18S and 25S rDNA genes, the internal transcribed spacer (ITS1 and ITS2), and the intergenic spacer 45S-IGS (25S-18S). This study identified six different types of 45S-IGS, A to F, which at 363 bp, 1121 bp, 1717 bp, 1969 bp, 2036 bp and 2111 bp in length, respectively, were much shorter than the reported reference IGS sequences in *B. oleracea* var. *alboglabra*. The shortest two IGS types, A and B, lacked the transcription initiation site, non-transcribed spacer, and external transcribed spacer. Functional behavior of those two IGS types in relation to rRNA synthesis is a subject of further investigation. The other four IGSs had subtle variations in the transcription termination site, guanine-cytosine (GC) content, and number of tandem repeats, but the external transcribed spacers of these four IGSs were quite similar in length. The 45S IGSs were found to follow Mendelian inheritance in a population of 15 F_1_s and their 30 inbred parental lines, which suggests that these sequences could be useful for development of new breeding tools. In addition, this study represents the first report of intra-specific (within subspecies) variation of the 45S IGS in *B. oleracea.*

## 1. Introduction

**Figure 1:**
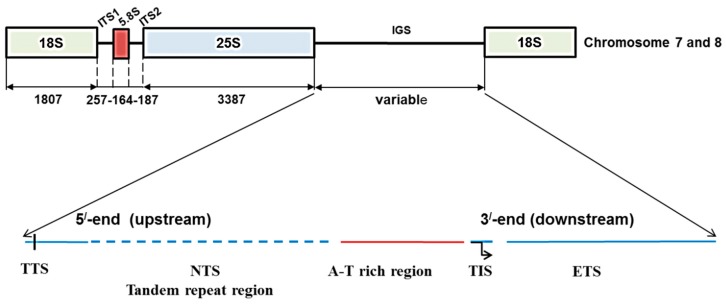
Ribosomal DNA array and organization of intergenic spacer (IGS) in *Brassica oleracea* based on Tremousaygue *et al.* [31] and the present study. Diagram shows position of 45S IGS region in between 25S and 18S rDNA subunits. TTS, transcription termination site; NTS, non-transcribed spacer; TIS, transcription initiation site and ETS, external transcribed spacer.

Ribosomes of living organisms are essential for protein synthesis, cellular growth, and organismal development. About 50% of all cellular transcription is modulated by ribosomal RNA (rRNA), which accounts for almost 80% of the total RNA in growing cells [1,2]. The presence of multiple copies of ribosomal DNA (rDNA) in eukaryotes in tandem units within the nuclear organizer region (NOR) of one or more chromosomes ensures the required level of ribosome supply [1,3]. Each rDNA repeat unit consists of coding regions for 18S, 5.8S, and 25S rRNAs, and three spacer sequences including an intergenic spacer (IGS) and two internal transcribed spacers, ITS1 and ITS2 [4,5]. The IGS contains the non-transcribed spacer (NTS) and external transcribed spacer (ETS) sequences [4,5,6] and the entire IGS region separates the 25S and 18S rRNA genes in each repeat unit (Figure 1) [5].

Together, the IGS represents a complex regulatory unit that includes repeating elements, repetitive enhancer elements, promoters and terminators of transcription, and a conserved secondary structure [4,5,6,7]. Whereas the coding regions of rDNA are highly conserved, the IGS regions display inter- and intra-specific polymorphism in both sequence and length [8,9,10,11,12,13]. In plants, the length of the IGS region in rDNA varies from 1 kb to more than 12 kb across different species from different genera [8,14]. Variability in the IGS region arises primarily from diversity in the numbers of internal sub-repeats arranged in the IGS. These internal sub-repeats, from 30 to 300 bp in length, contain duplicated core promoter in some eukaryotes [1,15,16,17,18,19]. Imbalanced or unequal crossing over within the repetitive sequences is probably responsible for the variation in copy number of the sub-repeats [17].

The variability in rDNA-IGS creates an opportunity to study interspecific and intraspecific evolutionary divergence [18]. Experimental evidence has suggested that the repeated elements of IGS might play a role in enhancing transcription in plants [20,21,22] and likely contain the signals for processing of pre-rRNAs [22,23,24]. These repeated elements may be involved in RNA polymerase I-mediated transcription [22,25]. The experimental results on the structure and sequence of the IGS also help explain the control of expression of rRNA genes.

The Brassicaceae family is characterized by the diverse nature of its member species and wide variation, even within species, in morphological and phytochemical characteristics. Cabbage (*Brassica oleracea* var. *capitata*), a subspecies within the Brassicaceae, is an agriculturally and economically important vegetable that is an integral component of the human diet in many countries of the world. Cabbage has a CC-type genome of approximately 648 Mbp in size, with *2n* = 18 [26,27,28]. Genetic variation in functional anti-carcinogenic and anti-oxidant components as well as phenotypic variation among the genotypes within the cabbage subspecies are well established in the literature [29]. The structure of rDNA in *B. oleracea* along with the intergenic spacer (IGS) is important for achieving high level signals of transcription and processing of rRNAs. In *B. oleracea*, there are two types of IGS, namely 5S IGS and 45S IGS (Figure 1). The 45S IGS is located in chromosomes C7 and C8 (Figure 1). The structure of IGS regions has been studied in species and subspecies of the Brassicaceae family [18,23,30,31,32,33,34]*.* The variability in IGS region has not been widely exploited, although it has been used for some analyses. For example, characterization of *Eruca*-*Brassica* hybrids using DNA probes was possible using rDNA of *E. sativa*, which is a wild relative of *Brassica* species that exhibits resistance to white rust and drought [17,35]. Studies related to IGS length variation have used both DNA gel blot hybridization and PCR. Each of the methods has both advantages and disadvantages; for example, DNA gel blot hybridization uses restriction enzymes and requires a larger amount of DNA initially, whereas PCR primer-based methods cannot amplify a DNA fragments larger than 4 kb but often selectively amplify smaller DNA fragments [19]. The existing IGS sequence of the *alboglabra* subspecies of *B. oleracea* in literature is based on DNA gel blot hybridization [30,31]. 

This study aimed to clone and sequence IGSs of cabbage lines by exploiting PCR-based techniques. We examined IGS length variation across a number of cabbage inbred lines and their hybrids to determine the amount of variation in this trait. This study also characterized the functional elements present in the IGSs, as well as their organization and secondary structure, and finally compared them with those of *B. oleracea* var. *alboglabra* and other crop plants. To fulfill these objectives, whole-genome sequencing of rDNA was conducted, and 5S and 45S IGS regions were cloned, sequenced and compared with reference gene annotations (45s IGS sequence of *B. oleracea* var. *alboglabra*). The variability observed in the 45S IGS was analyzed and the patterns of inheritance were studied in comparatively larger parent-hybrid populations.

## 2. Results

### 2.1. Identification of 45S Ribosomal DNA

Whole-genome sequencing generated approximately 6x coverage across four lines sequenced between 3675 Mbp and 4177 Mbp (Table S1). Further analysis of the rDNA region with PHYZEN software confirmed the presence of 45S IGS regions in chromosomes C7 and C8 (Figure 1). As the focus of this study was to explore the diversity in 45s IGS region based on PCR amplification, the whole-genome sequencing data were used as background information only. The 45S IGS region including 25S, 18S, 5.8S, ITS1, and ITS2 subunits accounted for 5802 bp in the four inbred lines. ITS1 and ITS2, measured 257 bp and 187 bp, respectively, and were also identical in the four inbred lines (Acc. KT377452 and KT377454). By contrast to the identical sequences of 5S IGS, ITS1 and ITS2; the 45S-IGS showed wider diversity (Figure S1).

### 2.2. Variation in 45S-IGS

**Figure 2:**
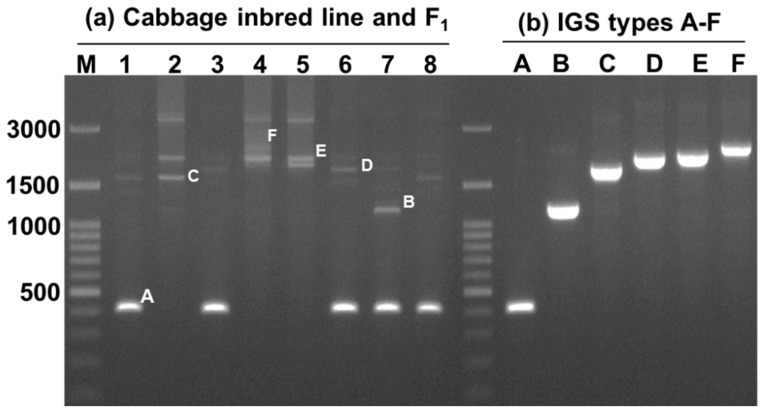
Different types of IGS (A to F types) identified in cabbage lines. (**a**) IGS regions identified in three cabbage inbred lines and five F_1_ plants and (**b**) cloned bands representing the six types of IGS amplified using specific PCR primers designed at the 25S-end and 18S-start regions.

**Figure 3:**
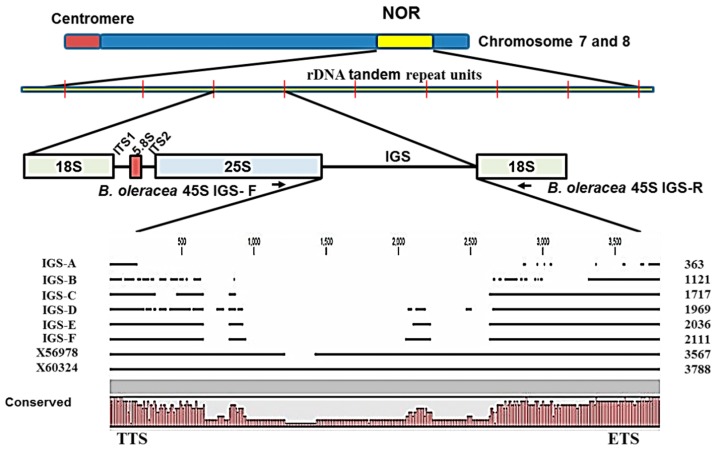
Six types of IGS (A to F) identified at the 25S–18S region after cloning and sequencing based on PCR primers designed at the end of 25S and the beginning of 18S. The 45S IGS region is located in rDNA tandem repeats of nucleolar organizer region (NOR) of chromosomes 7 and 8 of *B. oleracea*. The PCR amplified IGSs had high sequence similarity with the reference sequences X56978 and X60324 (45s IGS sequence of *B. oleracea* var. *alboglabra*). Transcription termination site (TTS) and external transcribed spacer (ETS) were highly conserved.

Cloning and sequencing of the entire 45S-IGS region in five F_1_ and three inbred lines revealed six different IGS types, which we named A to F (Figure 2). The product sizes of the 45S-IGS region ranged from 363 bp to 2111 bp (Figures S2 and S3; Acc. KT377443, KT377444, KT377445, KT377446, KT377447, KT377448). These six types of IGS had average sequence similarity of 95%, with the D type the most different compared to any other type (Figure 3 and Figure S2). All six types of IGS region were much shorter than that of *B. oleracea* var. *alboglabra* [30,31] which are 3567 bp and 3788 bp in length, respectively (Figure 3 and Figure S2A). The F-type IGS was the longest we identified, but was still 1447 bp shorter than X56978, the reference sequence of 45s IGS of *B. oleracea* var. *alboglabra* (Figure 3), whereas the A-type one, at only 363 bp, was the shortest (Figure 3 and Figure S2A). The B-type IGS was more than three times longer than the A-type one and its base sequences at the end of 25S were dispersedly distributed compared to the reference sequences (Figure 3 and Figure S1). The C-type IGS had an additional 596 bp compared to the B type, and this IGS was constituted of two comparatively smaller sequences at the end of 25S and one larger sequence at the beginning of the 18S end (Figure 3 and Figure S1). The D-type IGS, 252 bp longer than the C type, was characterized by the presence of some shorter sequences in the middle rather than at the two ends (Figure 3). In addition, its 25S end was much dispersed compared to the C, E and F types but less dispersed compared to the B type (Figure 3 and Figure S1). The most symmetry in sequence organization existed between the E- and F-type IGSs, and these two longer IGS types had only 75 bp differences (Figure 3).

A phylogenic tree revealed in two major clusters for the six types of IGS (Figure S2B). The D and B types, which have dispersed sequences at the 25S end, yielded one cluster, whereas the other four IGS types were in another cluster with the reference sequences (Figure S2B). In the larger cluster, the IGS types E and F formed a close sub-cluster that was similar to the type C. The smallest IGS, A type, was the most distant in that cluster (Figure S2B). The B-type IGS had the lowest guanine-cytosine (GC) percent of 39.5 and the F-type IGS had the highest, 46.8% (Table 1). In general, the GC content was higher at the 25S end of the IGS compared to the 18S end in both *B. oleracea* and *S. alba* (Table S2). The similarity in gene size and GC% between these two species supports the validity of our results.

**Table 1:** Best hit information from Basic Local Alignment Search Tool for nucleotides (BLASTn) of National Center for Biotechnolgoy Information (NCBI).

IGS Type	Description	Query Cover (%)	*E* Value	Identity	Accession	GC Content (%)
IGS_A	*B.oleracea* 25S & 18S rRNA genes	73	7 × 10^−85^	98%	X60324.1	44.08
IGS_B	*B.oleracea* 25S & 18S rRNA genes	44	0.0	98%	X60324.1	39.52
IGS_C	*B.oleracea* 25S & 18S rRNA genes	100	0.0	96%	X60324.1	46.53
IGS_D	*B.oleracea* 25S & 18S rRNA genes	85	0.0	87%	X60324.1	44.59
IGS_E	*B.oleracea* 25S & 18S rRNA genes	100	0.0	94%	X60324.1	46.37
IGS_F	*B.oleracea* 25S & 18S rRNA genes	100	0.0	95%	X60324.1	46.80

### 2.3. Functional Elements and Domains

**Figure 4:**
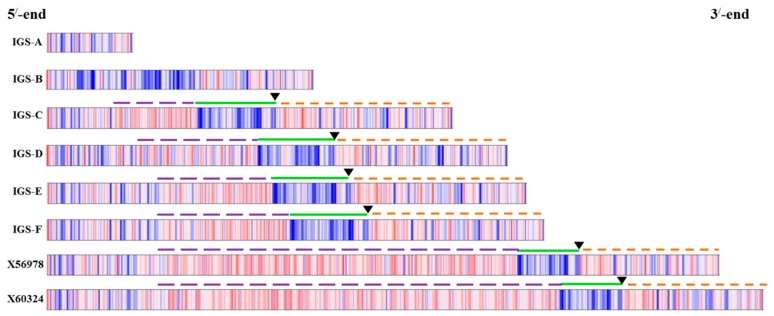
Functional elements and domains identified in the six IGS types. The transcription initiation site (TIS) of *B. oleracea* sequence is TATATAAGGGG (black down-arrow) for C, E, F and reference sequences X56978 and X60324, and that of the D type is TATATAAAGGG. Violet dashed line represents tandem repeat region, green line represents AT-rich region, orange dashed line represents ETS region, red and blue bars respectively represent the locations of GC and AT bases. The A and B types are devoid of tandem repeats, TIS and ETS. Transcription termination site is located at the beginning of 3’ end.

In recent studies, the IGS region has been investigated in detail in terms of its organization of functional elements and domains. CLC Main Workbench (http://www.clcbio.com/products/clc-main-workbench/) analyses indicated that tandem repeat regions exist in the C, D, E and F types of IGS (Figure 4). The A and B types of IGS were lacking in tandem repeat regions, TIS and ETS (Figure 4 and Figure S3) but the transcription termination sequence (TTS) was quite similar to those of the other four types of IGS (Table 2). The C to F types of IGS had similar 5’ETS sequences (738–744 bp in length, 46.3 to 49.8 GC%). Their adenine-thymine (AT) rich regions contained more than 68.9% AT, and TISs were adjacent to the AT rich regions (Figure 4, Table 2). However, the length of the repeat sequences of those four IGS types was highly variable (Figure 4, Table 2). The position of repeat sequences was 290–443 bp downstream from the TTS and that was always upstream from the TIS and ETS (Figure 4). The length of repeat sequence was 337, 511, 506 and 580 in C, D, E and F types of IGS, respectively, which were much shorter than those in the reference sequences (2040 and 2255 bp, respectively, in X56978 and X60324; Table 2). Sub-repeat sequences were 12 to 74 bp in length across the four IGS types with 84% to 97% identity among the sub-repeats (Table 2 and Table S3). By contrast, in the two reference 45S IGSs the length of sub-repeats ranged between 12 and 454 bp with 67%–97% identity (Table 2 and Table S3). The lengths of the AT rich regions of the C, E and F types of IGS were the same as in the reference sequences, 329 bp, except for the D-type IGS, which was 322 bp in that region (Table 2). The transcription initiation sequence (TIS) of the C–F IGSs was identical to that of the reference sequences (TATATAAGGGG) except the D type, which had TATATAA**A**GGG (Table 2). By contrast, the TTSs of the B, C and F types were identical, but one base different from the reference sequences whereas the A-type IGS had an identical TTS to the reference sequences (Table 2). The E-type IGS was one base shorter in the TTS compared to any other type including the references (Table 2). The NTS regions of the C–F types were much shorter compared to the reference sequences (Figure 4). The C-type IGS bore a shorter NTS compared to the other three types (Figure 4). All six IGS types shared 94% or higher similarity with accession number X60324, except the type D which shared 87% sequence similarity (Table 1). Notably, types C, E and F had 100% query coverage with that reference sequence (Table 1).

**Table 2:** Annotation of variation in 45S-IGS region.

IGS Type	TTS Sequence and Position	Repeat Position	Consensus Size of Tandem Repeats (bp)	Percent Match	AT-rich Position (AT%)	TIS Sequence and Position	ETS Size (GC %)
IGS_A	CCCTCCCCCTTTCT (1–14)	None			None	None	None
IGS_B	CCCTCCCCCTTTCC (1–14)	None			None	None	None
IGS_C	CCCTCCCCCTTTCC (1–14)	304–640	12–72	85–96	634–962 (69.91)	TATATAAGGGG (963–973)	744 (49.19)
IGS_D	CCCTCCCCCATTCC (1–14)	395–905	19–58	85–97	899–1220 (68.94)	TATATAAAGGG (1221–1231)	738 (46.34)
IGS_E	CCCTCCCCTTTCC (1–13)	454–959	12–72	84–96	953–1281 (69.60)	TATATAAGGGG (1282–1292)	744 (49.19)
IGS_F	CCCTCCCCCTTTCC (1–14)	455–1034	12–74	84–96	1028–1356 (69.60)	TATATAAGGGG (1357–1367)	744 (49.19)
X56978	CCCTCCCCCTTTCT (1–14)	456–2495	12–454	68–96	2489–2817 (70.21)	TATATAAGGGG (2818–2828)	739 (49.80)
X60324	CCCTCCCCCTTTCT (1–14)	457–2711	12–250	67–97	2705–3033 (70.21)	TATATAAGGGG (3034–3044)	744 (49.33)

Repeat position was obtained using tandem repeat finder (http://tandem.bu.edu/trf/trf.html) comparing with accession no. X60324 of NCBI. AT-rich was identified using CLC Main Workbench 7.5.1.

### 2.4. Variable IGS Inheritance

**Figure 5:**
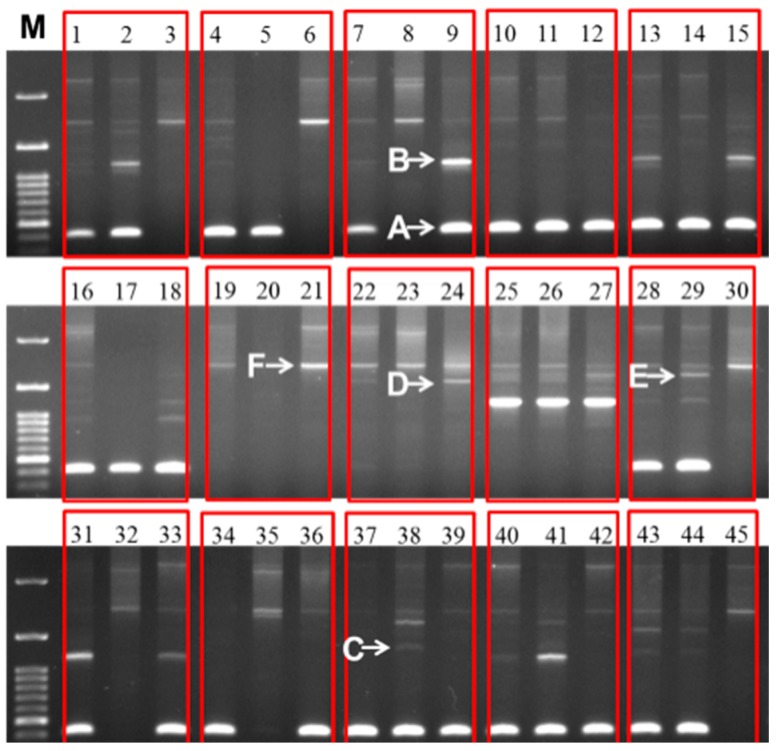
Different sizes of 45S (25S-18S) IGS sequences observed in 30 inbred lines of cabbage and their 15 F_1_ progeny. The line numbers correspond to those in Table 3C. Within each red box the first, second, and third lanes represent F_1_, female parent, and male parent, respectively. M, 100 bp ladder.

We explored the inheritance of IGS sequence because our above findings of variability within subspecies suggested that this might open a new window of using IGS as a breeding tool. When the heredity of IGS was tested in a population consisting of 15 F_1_ lines and their 30 parents, all progeny were found to harbor the parental-type IGSs (Figure 5). For eight out of 15 F_1_ samples, the male and female parents had similar IGSs and the progeny contained the same type (Figure 5). Four F_1_ progeny produced the male parental type (nos. 1, 4, 28 and 43 in Table 3C) IGS, and three F_1_s progeny produced the female parental type IGS (nos. 7, 31 and 34 in Table 3C) (Figure 5). Out of 45 parents and F_1_ progeny, the A-type IGS was found in 29 lines, although some of them had other IGS types coupled with the A type (Figure 5). The A type of IGS was present in F_1_ progeny 9051 × 3074 (no. 10 in Table 3C), 2409 × 8S8-7 (no. 13), 26S × NC1 (no. 16), 842 × 2409 (no. 37), and 496B × 2409 (no. 40) with both of their parents (Figure 5). The F_1_ 2409 × 8S8-7 (no. 13) and its female parent, F_1_ 832 × 755 (no. 25) and both parents, and F_1_ 337S × 94 (no. 31) and its female parent had the B type of IGS (Figure 5). The F_1_ 832 × 755 (no. 25) and both of its parents bore both C- and D-type IGSs (Figure 5).

## 3. Discussion

Sequencing of ribosomal spacers involves some difficulties. Firstly, the IGS is a highly folded non- protein-coding region and so is not comparable to commonly used protein or cDNA sequences. Secondly, rDNA units are highly repeated (Figure 4). Thirdly, the IGS sequence is unusually GC-rich. Moreover, next-generation sequencing can sequence 45S rDNA only by excluding its IGS region. For these reasons, we chose PCR-based cloning and sequencing in this study to determine IGS sequences. Interestingly the lengths of all six types of 45S IGS we identified (Figure 2 and Figure 3) were much shorter than the reference sequences GenBank: X56978 [30] and GenBank: X60324 [31]. In addition, one or a few more additional IGS other than the six identified are possible those are larger than 3000 bp (Figure 5). The longest, F-type IGS identified in this study was 1456 bp and 1677 bp shorter than X56978 and X60324, respectively. Both Tremousaygue *et al.* [30] and Bennett and Smith [31] conducted restriction enzyme-based cloning in *B. oleracea* var. *alboglabra*, which is dependent on the recognition site whereas our study was based on specifically-designed PCR primers. Therefore, the differences in IGS sequences are probably partly explained by methodical differences and partly by subspecies differences. It is important to note that the restriction enzymes used in those two studies, EcoR1 and BamH1, also digest some portion of 25S and 18S sequence along with 25S-18S IGS. By contrast, one of the disadvantages of PCR primer-based cloning is selective amplification of small DNA fragments. Therefore, further confirmation is required for the existence of the two smallest types we identified, the A and B types, as these two IGS were devoid of any repeated elements, the ETS and the TIS (Figure 4) even though they had TTS sequence (Table 2). However, nucleotide comparison through BLAST search with NCBI reference sequence confirmed 98% identity for both A and B types compared with reference X60324 of *B. oleracea* var. *alboglabra* [31]. It is possible that these two IGSs were subject to large deletions at their downstream end during evolution.

Sequences of IGS regions are also difficult to elucidate because as one individual cell may retain more than one type of IGS (Figure 2 and Figure 5). Tremousaygue *et al.* [31] published an updated sequence of the IGS region of *B. oleracea* var. *alboglabra* after Bennett and Smith [30] which is 206 bp longer. That variation is explained by the different digestion sites of EcoR1 used by [31] and BamH1 used by [30]. However, Tremousaygue *et al.* [31] marked internal sequence variation in sequence with that of Bennett and Smith [30] as errors associated with reading errors. In our study, several PCR conditions have been tested and obtained similar bands. The other way to confirm the size of the IGS is to digest total DNA with a restriction enzyme which does not cut within the IGS and to hybridize with the appropriate probe. Such an experiment would further validate the size of the IGS and would confirm its variability.

Intra-specific length and sequence heterogeneity among different types of IGS, sometimes even within an individual plant, as observed in our study (Figure 2 and Figure 3) is a common feature of IGSs in almost all plant species studied. Heterogeneity of IGS in Brassicaceae family was first reported in *Arabidopsis* [36,37]. During evolutionary development of species, any insertion or deletion of AT-rich or GC-rich sequences in the tandem repeat region may also accelerate the length heterogeneity. Other probable causes are deletions, possibly reflected in the A- and B-type IGSs of our study, or duplications of the sub-repeat regions [38,39,40] or of the ETS [41,42], and duplications of the promoter region [32,39]. The 5*’*ETS length observed in this study was consistent with those reported for *B. oleracea alboglabra* and *Raphanus sativa* [43], while widely variable ETS lengths were recorded in other plant species, for example, 5.8 kb in *Medicago* [44], 1227 to 1735 bp in *Oryza* [45], 1155 to 2226 bp in *Vigna* [25] and 529 to 823 bp in *Zea* [46].

The length and occurrence of sub-repeats is another source of variation in IGS sequences (Table S3, Figure S3). The length of the repeat elements in our study ranged between 12 to 74 bp in C- to F-type IGSs (Table 2). In some other plant species, the repeat region is much longer, for instance, 130 bp in wheat [47], 200 bp in maize [16], 325 bp in *Vicia faba* [15], and 460 bp in carrot [48]. In potato, the length of sub-repeats ranged between 20 and 56 bp [49]. Similar to *E. sativa* [17], *Arabidopsis* [32], and *Sisymbrium irio* [50], we found repeat families downstream of the putative TTS in cabbage (Figure 4). 

The presence of a TATA box in the core promoter of TIS was the characteristic feature of *B. oleracea* var. *capitata* 45S IGS (Table 2), similar to those of *B. oleracea* var. *alboglabra* [30,31] and *Medicago* [43,51]. In the C–F types of IGS, the presence of motifs and domains putatively associated with initiation and termination of rRNA transcription and the presence of rDNA promoters, promoter/enhancer elements and TATA box involved in the transcription by RNA polymerase I, indicated that the structural variants of IGS possess the necessary regulatory elements to be functional [1,20,25,32,39,45,52,53,54]. In addition, the identical sequence of TIS for most of the identified IGS indicated that this sequence is generally conserved (Table 2).

**Figure 6:**
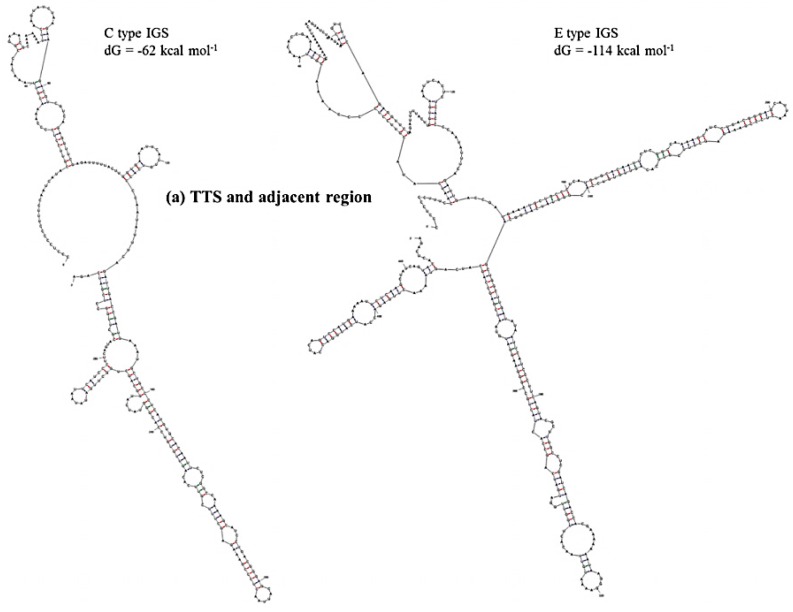

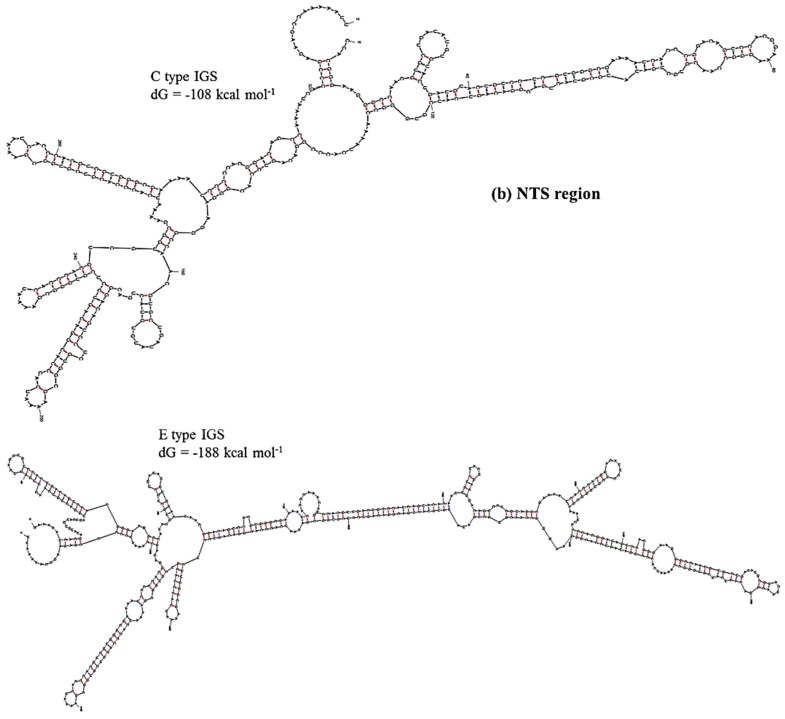
Predicted secondary structures of (**a**) TTS and adjacent region (Region 1) and (**b**) NTS region (Region 2) of C and E type 45S IGS of *B. oleracea* var. *capitata* and their minimum free energies.

Prediction of secondary structure and associated *dG* values can indicate at what level IGS sequences are active in producing ribosomes for the biosynthesis of proteins. In secondary structure prediction, mfold estimates free-energy as *dG* value. The predicted secondary structures and their free energies, as calculated using our results, indicated that the IGS regions form extensive and strong secondary structures with negative free-energy to start spontaneous production of protein. Free energy is available for transcriptional regulation by the non-coding RNA transcripts, initiation of replication at the AT-rich region and production of pre-rRNA transcript at the 5’ETS region. We divided the IGS sequences of the C and D types into four regions: Region 1, TTS and adjacent region; Region 2, NTS region; Region 3, AT-rich region and Region 4, TIS and 5’ETS region (Table 4). Free energies predicted as *dG* at Region 3 (−46 to −54 kcal·mol^−1^) and Region 4 (−273 kcal·mol^−1^ except D-type IGS) were quite similar for the four IGS types and the reference sequences X56978 and X60324 (Table 4). Region 1 was predicted to have much lower *dG* for E- and F-type IGSs compared to C- and D-type IGSs. The E-type IGS was predicted to form four additional loops in its secondary structure at Region 1 compared to the C-type IGS (Figure 6). The predicted *d*G value greatly differed at Region 2, the sub-repeat region, among the four IGS types (Table 4). The C-type IGS had an 80 kcal·mol^−1^ higher *dG* value that predicted three fewer loops compared to the E type (Figure 6). Low-energy secondary structure models suggest that with their low free-energy levels, the highly conserved structures, for example 5’ETS, possibly act as evolutionary constraints [55]. 

Out of 15 F_1_ lines, none produced any intermediate or off-parental type offspring. These results are suggestive of complete dominance of the variable IGS (Figure 5). However the issue should be further examined in subsequent segregating generations for confirmation. Our preliminary results suggest that IGS sequence could potentially be used as molecular markers in breeding programs, especially in selection of desired hybrids from a large number of crosses.

## 4. Materials and Methods

### 4.1. Plant Materials and DNA Extraction

Seed of three different sets of *B. oleracea* L. var. *capitata* subspecies was purchased from Asia Seed Co., Ltd. (Seoul, Korea) (Table 3). A set of four inbred cabbage lines was used for whole-genome sequencing (Table 3A), a set of eight genotypes (three inbred lines and five F_1_s) was used to study the 5S and 45S IGSs, as well as ITS1 and ITS2 after cloning and sequencing (Table 3B), and a set of thirty inbred lines and their fifteen F_1_ hybrids were used to test the inheritance pattern of 45S IGS (Table 3C).

Plants were raised under fluorescent lights in a growth chamber for 30 days before leaf samples were collected from theselected plants of each parental line or their F_1_ progenies. The seedlings were raised in garden soil mixed with peat moss, coco peat, perlite, zeolite, and vermiculite. The fresh leaves were snap frozen with liquid nitrogen and stored at −80 °C until DNA extraction. Genomic DNA of all cabbage genotypes was isolated according to the instructions provided by the manufacturer by using the DNeasy Plant Mini Kit (QIAGEN, Hilden, Germany). Quality and quantity of DNA extracted were monitored by electrophoresis on 1% gel agarose with undigested lambda DNA and confirmed using a Nanodrop ND-1000 spectrophotometer (NanoDrop, Wilmington, DE, USA).

### 4.2. Whole-Genome Sequencing

Whole-genome sequences of the selected genotypes were acquired via next-generation sequencing (NGS) using the Illumina HiSeq platform (Illumina, San Diego, CA, USA). The sequences obtained after NGS were used to design primers for the ITS1 and ITS2 and IGS regions. Ribosomal DNA was analyzed using PHYZEN (http://phyzen.com).

### 4.3. PCR Amplification Cloning and Sequencing

Primers were designed to clone the 45s IGS at the 3′ end of 25s rRNA subunit and 5′ end of 18s rRNA subunit based on published reference sequences of *Sinapsis alba* (Accession nos. 5S: X56866 [56]; 45S: X66325 [57]) and those were also compared with our whole genome sequencing data (Table 5, Figure 1). Primer3 software was used for designing primers (http://primer3.ut.ee/; Table 5). Primers were also designed to clone 5S IGS, ITS1 and ITS2 following the reference sequences of *Sinapsis alba* (Table 5) [56,57]. Two different intergenic primers were required to develop for D type 45s IGS and C, E and F types 45s IGS as the cloning kit used was only able to clone a sequence smaller than 1000 bp at a time, at the downstream of 3′ end of 25s rRNA subunit and at the upstream of 5′ end of 18s rRNA subunit (Table 5). Both D type and CEF type intergenic primer sequences were designed approximately 900 bp apart from the 3′ end of 25s rRNA subunit. Emerald PCR master mixture (Takara, Shiga, Japan) was used for PCR amplification of rDNA. PCR amplifications were carried out by incubation at 95 °C for 3 min followed by 35 cycles of denaturation, annealing and extension. Denaturation was carried out at 95 °C for 1 min, primer annealing at 55 °C for 1 min and primer extension at 72 °C for 1 min, followed by a final extension step of 5 min at 72 °C in an Eppendorf thermal cycler (Eppendorf AG, Hamburg, Germany). The PCR products were visualized on 1% agarose gels as a single band with a 100 bp ladder DNA. The amplified DNA fragments were purified using Promega Purification kit (Promega, Madison, WI, USA) according to the manufacturer’s instructions. Cloning was performed using the TOPO TA cloning kit (Invitrogen, Carlsbad, CA, USA). The cloning was conducted following the manufacturer’s instructions but the reaction was downscaled to half. Three clones were selected for sequencing against each IGS type. The cloned DNAs were sequenced with the universal primers M13F and M13RpUC using the ABI 3730XL sequencer (Macrogen Co., Seoul, Korea). To eliminate ambiguities, each sequencing reaction was repeated three times, especially in places where there were no reverse primers. Sequences obtained were initially compared with reference sequence *Sinapsis alba* (Accession nos. 5S: X56866 [56]; 45S: X66325 [57]) to compare gene size and GC content as high similarity exists for rDNA between *B. oleracea* and *S. alba*. The 45S IGC sequences were then analyzed using CLC Main Workbench version 7 (CLC, Aarhus, Denmark) coupled with NCBI reference accessions X56978 and X60324 of *B. oleracea* var. *alboglabra*.

**Table 3:** Plant materials obtained from the Asia Seed Co., Ltd. (Seoul, Korea). Materials were used to (**A**) study whole-genome sequence of rDNA through next-generation sequencing (NGS); (**B**) clone and sequence ITS1, ITS2, and IGS regions; (**C**) test 45S IGS variation in 30 inbred lines and their 15 F_1_s, F = female parent line, M = male parent line.

(A) Inbred Lines for NGS		(B) F_1_ Combination or Parents			(C) Lines and F_1_s to Test 45S IGS Variation								
Sl. no.	Line	Sl. no.	Lines or cross combinations	Type	Sl. No.	Lines or cross combinations	Type	Sl. No.	Lines or cross combinations	Type	Sl. No.	Lines or cross combinations	Type
1	Asiaseed-14	1	8352 × 636	F_1_	1	1464 × 621	F_1_	16	26S × NC1	F_1_	31	337S × 94	F_1_
2	Asiaseed-17	2	2418 × CT5-51	F_1_	2	1464	F	17	26S	F	32	337S	F
3	Asiaseed-28	3	842 × 621	F_1_	3	621	M	18	NC1	M	33	94	M
4	Asiaseed-31	4	94	Inbred line	4	8352 × 636	F_1_	19	337 × P15-41	F_1_	34	IB14 × DWB	F_1_
		5	IB14	Inbred line	5	8352	F	20	337	F	35	IB14	F
		6	842	Inbred line	6	636	M	21	P15-41	M	36	DWB	M
		7	496B × 2409	F_1_	7	225 × 95	F_1_	22	2418 × CT5-51	F_1_	37	842 × 2409	F_1_
		8	9051 × PKT-41	F_1_	8	225	F	23	2418	F	38	842	F
					9	94	M	24	CT5-51	M	39	2409	M
					10	9051 × 3074	F_1_	25	632 × 755	F_1_	40	496B × 2409	F_1_
					11	9051	F	26	632	F	41	496B	F
					12	3074	M	27	755	M	42	2409	M
					13	2409 × 8S8-7	F_1_	28	842 × 621	F_1_	43	9051 × PKT-41	F_1_
					14	2409	F	29	842	F	44	9051	F
					15	8S8-7	M	30	621	M	45	PKT-41	M

**Table 4:** Maximum negative *dG* (predicted free energies, kcal·mol^−1^) values obtained during putative secondary structure formation in four different regions (R) of 45S IGS.

45S IGS	TTS + 3’ETS Region, Region 1	NTS Region, Region 2	AT-rich Region, Region 3	5’ETS Region, Region 4
C type	−62	−108	−48	−273
D type	−67	−171	−46	−239
E type	−114	−188	−58	−273
F type	−113	−209	−58	−273
X56978	−110	Not obtained	−54	−277
X60324	−107	Not obtained	−54	−276

**Table 5:** Primers designed using Primer3 software to identify 5S and 45S (25S-18S) IGS, ITS1, and ITS2 of *B. oleracea* var. *capitata*.

Name	Forward and Revers Primers	Product Size	Comment
Bo 5S IGS	F: ACTAGGATGGGTGACCTCCCG;	484	5S-IGS product size
	R: CGCTTAACTGCGGAGTTCTGA		
Bo ITS1 and 2	F: CGCGAGAAGTCCACTAAACC;	823	Identify sequence differences
	R: ACGTCCGATTTTCAAGCTGG		
Bo 45S IGS	F: ATTCAGCCCTTTGTCGCTAA;	variable	45S-IGS product size
	R: ATGACTACTGGCAGGATCAACCAG		
IGS inter-D	ATCGTGTAGGACCGAGAAGT		D type intergenic
IGS inter-CEF	GGTAGGCACAAAAGGAATGC		C, E and F type intergenic

### 4.4. Sequence Analysis

The entire IGS region was assembled into a contig file by aligning the overlapped sequences of DNA. The sequences were analyzed using CLC Main Workbench version 7 to identify functional elements and domains of IGS, e.g., ETS, NTS and AT-rich regions. The transcription initiation site (TIS) and transcription termination site (TTS) were compared with those of *Fagus* and *Quercus* [58]. Multisequence alignments were generated by the program mVISTA using the ClustalW method [59] with gap opening penalty of 15 and gap extension penalty of 6. Repeat positions in the identified 45S IGS and NCBI reference sequences X56978 and X60324 were determined using tandem repeat finder (http://tandem.bu.edu/trf/trf.html) and the results were prepared using Dot matrix view software from NCBI after a BLAST search [60]. CLC Main Workbench version 7 software was used to generate the maximum-likelihood phylogeny trees tools. The Jukes-Cantor nucleotide distance measure was used for neighbour-joining. Finally, the phylogeny trees were generated using Molecular Evolutionary Genetics Analysis version 6 (MEGA6, http://www.megasoftware.net/) [61]. The secondary structure of 45S IGS was predicted using the mfold Web Server (http://mfold.rna.albany.edu/?q=mfold). The following accession numbers: KT377443–KT377455 were obtained after submitting the nucleotide sequences to the GenBank database.

## 5. Conclusions

The objective of this study was to examine the rDNA sequence in *B. oleracea* var. *capitata* coupled with investigating any variability in 45S IGS sequence (25S-18S IGS) based on PCR-based sequencing. There was no variation in ITS1 and ITS2 sequences in four inbred cabbage lines based on next-generation sequencing. However, our PCR-based cloning and sequencing identified six heterogeneous IGS types. Two IGS types were devoid of any sub-repeats, TISs and ETSs suggesting a major deletion in 45S IGS, but these two IGSs had putative TTSs and BLASTn searches showed up to 98% sequence identity with *B. oleracea* var. *alboglabra*. The other four IGS types had comparable ETS, TIS and TTS regions with subtle variation. The length of the repeat region of these four IGS types were remarkably shorter than reference sequences, which is consistent with the strikingly lower number of sub-repeats present in the identified IGSs. The 45S IGS regions identified here in cabbage were polymorphic in 15 F_1_ lines and their 30 inbred male and female parental lines. The IGS fragments in the F_1_ generation were of either male or female parental type. The inheritance pattern of IGS supports their potential uses in breeding as molecular markers. Variation of IGSs between cabbage lines and the NCBI reference IGS of *B. oleracea* var. *alboglabra* and the existence of polymorphic IGS fragments within genotypes highlight the need for further examination of IGS sequence in *B. oleracea*. Moreover, the functionality of variable IGS types in rRNA synthesis is a subject of further investigation.

**Table:** GenBank accession numbers:

KT377443	45S intergenic spacer (A type)	PCR-primer amplified
KT377444	45S intergenic spacer (B type)	
KT377445	45S intergenic spacer (C type)	
KT377446	45S intergenic spacer (D type)	
KT377447	45S intergenic spacer (E type)	
KT377448	45S intergenic spacer (F type)	
KT377450	5S intergenic spacer	
KT377452	Internal transcribed spacer 1	
KT377454	Internal transcribed spacer 2	

## Supplementary Materials

Supplementary materials can be found at http://www.mdpi.com/1422-0067/16/12/26125/s1.

## Abbreviations

rDNA: Ribosomal DNA; IGS: Intergenic spacer; ETS: External transcribed spacer; ITS: Internal transcribed spacer; NOR: Nucleolus organizer region; NTS: Non-transcribed spacer; TIS: Transcription initiation site; TTS: Transcription termination site; PCR: Polymerase chain reaction.
