# Supplementary material for: Diversity and Inheritance of Intergenic Spacer Sequences of 45S Ribosomal DNA among Accessions of Brassica oleracea L. var. capitata
Source: Int J Mol Sci. 2015 Dec 3;16(12):28783–99. doi: 10.3390/ijms161226125 (PMC4691072; doi:10.3390/ijms161226125)
Supplement: Supplementary file 1 [file ijms-16-26125-s001.pdf]

Supplementary Materials: Diversity and Inheritance of Intergenic Spacer Sequences of 45S Ribosomal DNA among Accessions of *Brassica oleracea* L. var. *capitata*

Kiwoung Yang, Arif Hasan Khan Robin, Go-Eun Yi, Jonghoon Lee, Mi-Young Chung, Tae-Jin Yang and Ill-Sup Nou

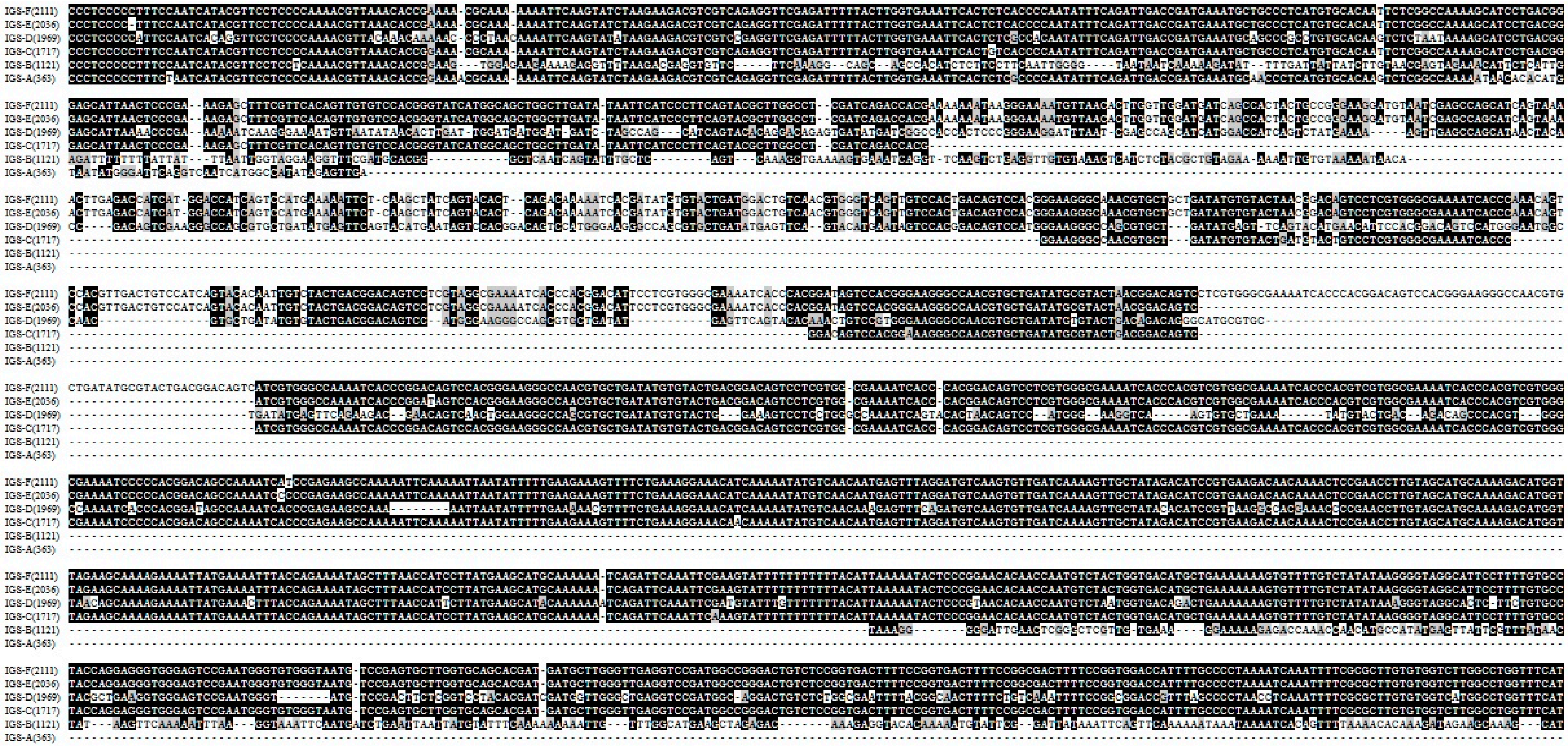

Figure S1. *Cont.*

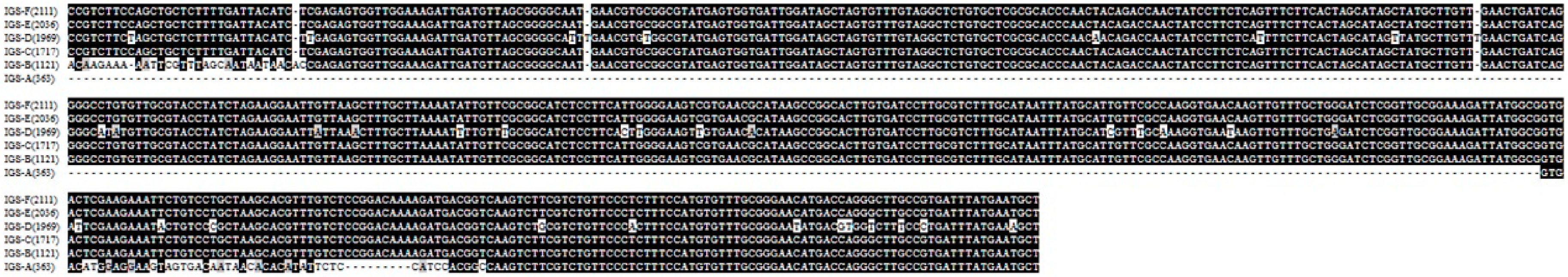

Figure S1. Six different types of IGS sequence as compared using Bio-edit at NCBI. Identical sequences are indicated by a black background, while >50% similarities are indicated by a grey background.

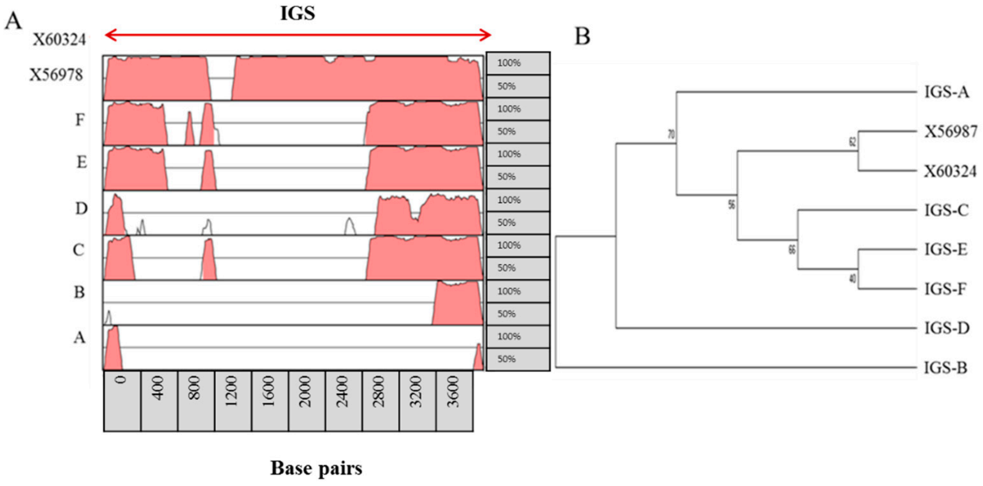

Figure S2. Comparison of the 45S IGS region using mVISTA program: (**A**) X60324 compared with X56978 showed ~200 bp in/del, E compared with F had ~73 bp in/del, C compared with E had ~315 bp in/del, A, B, and D were more heterogeneous; (**B**) phylogenetic tree. The reliability value for each internal branch indicates in percent how often the corresponding cluster was found among 1000 intermediate trees.

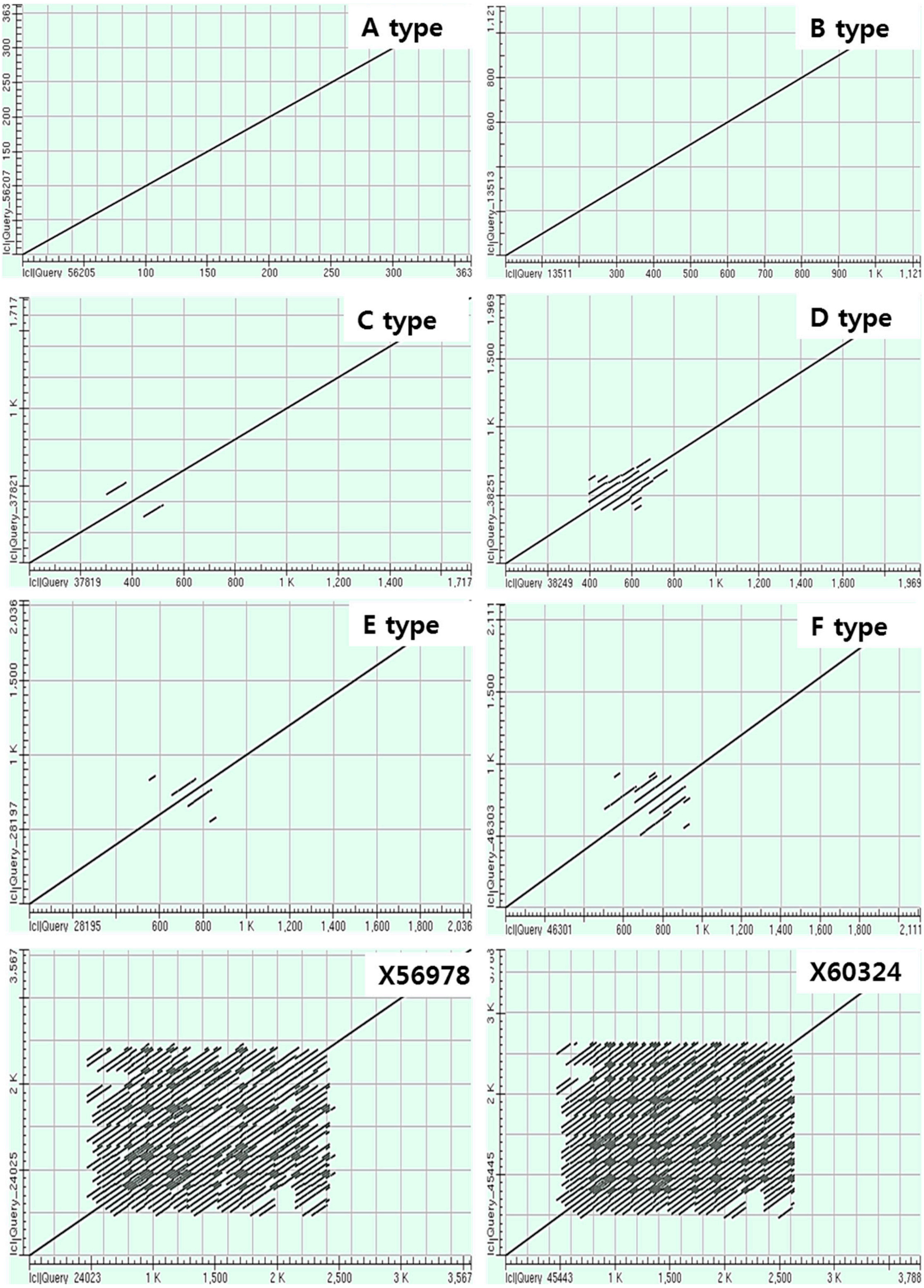

Figure S3. Dot matrix self-comparison plot of the *B. oleracea* var. *capitata* IGS sequences. **A** diagrammatic representation of the intergenic spacer is shown above the plot. IGS-A and IGS-B did not show repeat region; **C**–**F** had from one to six tandem repeat sequences. X56978 and X60324 are two reference sequences available in NCBI.

Table S1. Whole-genome sequencing for 5S and 45S rDNA annotation.

Inbred Line Name	Read Count	Total Bases (bp)	Coverage
Asiaseed-14	38,709,840	3,909,693,840	6.2x
Asiaseed-17	36,389,648	3,675,354,448	5.8x
Asiaseed-28	41,360,398	4,177,400,198	6.6x
Asiaseed-31	37,437,444	3,781,181,844	6.0x

Table S2. Comparison of different rDNA sub-units between *S. alba* 5S [Acc. No. X56866 (Capesius 1991a)] and 45S [X66325 (Capesius 1991b)] with those of *B. oleracea* (next-generation sequencing data of the present study).

Genes	*S. alba*		*B. oleracea*		Difference Count (bp)
	Gene Size (bp)	GC Contents (%)	Gene Size (bp)	GC Contents (%)	
5S	118	57.63	118	57.63	0
5S-IGS	373	40.48	385	42.60	122
45S-18S	1804	49.00	1807	49.31	23
45S-ITS1	265	50.57	257	51.75	35
45S-5.8S	163	52.76	164	53.66	4
45S-ITS2	188	54.79	187	54.55	18
45S-25S	3381	55.19	3388	55.40	60

Table S3. Sub-repeat traits of 45S IGS in *B. oleracea* var. *capitata*.

Indices	Period Size	Copy Number	Consensus Size	Percent Matches	Percent Indels	Score	A	C	G	T	Entropy (0-2)
**(a) C Type**											
304–522	72	3.0	72	90	2	350	25	27	29	17	1.97
483–544	30	2.1	30	96	3	117	27	33	25	12	1.93
523–608	21	4.0	22	95	3	149	26	33	25	13	1.94
532–625	42	2.2	41	85	7	118	31	36	20	11	1.88
1090–1132	12	3.6	12	93	0	77	6	27	27	37	1.83
**(b) D Type**											
395–590	58	3.4	58	97	0	356	27	20	30	20	1.98
557–696	44	3.1	44	78	6	172	26	20	32	20	1.97
858–902	20	2.4	19	85	14	58	44	33	15	6	1.73
**(c) E Type**											
626–696	30	2.4	30	90	0	115	28	32	23	15	1.95
656–830	72	2.4	72	90	2	280	26	27	28	17	1.97
802–863	30	2.1	30	96	3	117	27	33	25	12	1.93
842–927	21	4.0	22	95	3	149	26	33	25	13	1.94
851–940	42	2.1	41	84	8	110	32	34	21	12	1.90
1409–1451	12	3.6	12	93	0	77	6	27	27	37	1.83

Table S3. *Cont.*

Indices	Period Size	Copy Number	Consensus Size	Percent Matches	Percent Indels	Score	A	C	G	T	Entropy (0-2)
**(d) F Type**											
627–697	30	2.4	30	90	0	115	28	32	23	15	1.95
657–916	74	3.6	74	93	2	445	26	28	29	15	1.96
877–938	30	2.1	30	96	3	117	27	33	25	12	1.93
917–1002	21	4.0	22	95	3	149	26	33	25	13	1.94
926–1016	42	2.2	41	84	7	112	32	34	20	12	1.90
1484–1526	12	3.6	12	93	0	77	6	27	27	37	1.83
**(e) Accession X56978**											
510–796	71	4.0	72	76	8	303	26	26	27	19	1.99
767–867	30	3.4	30	90	0	166	27	31	24	15	1.96
703–1064	134	2.7	134	94	1	640	25	28	29	16	1.96
901–1001	30	3.4	30	95	0	184	27	32	25	13	1.94
827–1279	205	2.2	205	95	2	818	26	29	28	15	1.96
961–1146	71	2.5	72	93	5	320	25	29	30	15	1.95
1106–1199	30	3.1	30	96	0	179	27	32	26	12	1.93
1239–1309	30	2.4	30	92	0	115	23	32	26	16	1.96
1269–1527	72	3.5	73	93	2	423	25	27	31	16	1.96
1166–1703	248	2.2	250	95	2	994	24	28	30	16	1.97
1487–1557	30	2.4	30	92	0	115	23	32	26	16	1.96
1517–1981	72	6.5	72	74	9	436	25	27	29	17	1.97
1663–1763	30	3.4	30	95	0	184	27	32	25	13	1.94
1487–1898	206	2.0	206	92	1	684	25	28	28	17	1.97
663–1970	454	2.8	454	68	21	550	25	27	29	16	1.97
1941–2011	30	2.4	30	95	0	124	25	32	26	15	1.95
1166–2319	176	6.6	176	78	9	962	25	27	29	16	1.97
1971–2154	68	2.5	73	84	11	252	26	29	27	16	1.97
2114–2184	30	2.4	30	92	0	115	28	33	23	14	1.94
2048–2256	104	2.1	98	85	7	278	25	29	26	17	1.98
2144–2402	72	3.6	74	86	3	366	26	27	28	17	1.97
1978–2424	248	1.8	247	90	3	720	26	28	28	17	1.97
2362–2424	30	2.1	30	96	0	117	26	31	26	14	1.95
2409–2463	19	2.8	20	88	2	85	34	32	18	14	1.91
2432–2492	19	3.2	19	82	11	63	44	32	14	8	1.75
2944–2984	12	3.4	12	96	0	73	7	31	26	34	1.84

Table S3. *Cont.*

(e) Accession X60324											
**Indices**	**Period Size**	**Copy Number**	**Consensus Size**	**Percent Matches**	**Percent Indels**	**Score**	**A**	**C**	**G**	**T**	**Entropy (0-2)**
512–1345	72	12.0	72	67	13	328	26	28	28	16	1.97
770–870	30	3.4	30	90	0	166	27	31	24	15	1.96
904–1004	30	3.4	30	97	0	193	27	33	25	12	1.93
1110–1210	30	3.4	30	97	0	193	27	33	25	12	1.93
1170–1355	72	2.6	74	95	4	351	25	29	30	14	1.95
1315–1415	30	3.4	30	95	0	184	27	32	25	13	1.94
830–1489	205	3.2	208	97	1	1249	26	29	29	15	1.96
934–1591	104	6.4	104	72	14	592	26	29	29	15	1.96
1449–1519	30	2.4	30	92	0	115	23	32	26	16	1.96
1479–1737	72	3.5	73	93	2	432	24	27	31	16	1.96
1375–2532	176	6.6	176	80	8	1039	25	27	29	16	1.97
1375–1913	248	2.2	250	97	1	1028	25	27	30	16	1.97
1697–1767	30	2.4	30	92	0	115	23	32	26	16	1.96
1727–2191	72	6.5	72	75	9	445	25	27	29	17	1.97
1873–1973	30	3.4	30	95	0	184	27	32	25	13	1.94
1697–2108	206	2.0	206	92	1	693	25	28	28	17	1.97
2151–2221	30	2.4	30	95	0	124	25	32	26	15	1.95
2079–2293	104	2.1	101	92	3	351	26	27	29	16	1.97
2181–2367	72	2.6	74	90	3	306	26	28	28	16	1.96
1901–2637	248	3.0	244	86	4	865	26	27	29	17	1.97
2327–2397	30	2.4	30	92	0	115	28	33	23	14	1.94
2255–2469	104	2.1	103	87	5	303	26	28	27	17	1.97
2357–2615	72	3.6	74	86	3	375	26	27	29	17	1.97
2575–2637	30	2.1	30	96	0	117	26	31	26	14	1.95
2616–2679	21	3.0	21	93	2	110	28	31	25	15	1.96
3160–3200	12	3.4	12	96	0	73	7	31	26	34	1.84
